# Willingness to accept malaria vaccines amongst women presenting at outpatient and immunization clinics in Enugu state, Southeast Nigeria

**DOI:** 10.1186/s12936-024-04914-1

**Published:** 2024-04-25

**Authors:** Awoere T. Chinawa, Edmund N. Ossai, Vivian O. Onukwuli, Obinna C. Nduagubam, Ndubuisi A. Uwaezuoke, Chinyere N. Okafor, Josephat M. Chinawa

**Affiliations:** 1 Department of Community Medicine, College of Medicine ESUT, Enugu, Nigeria; 2 Department of Community Medicine, Ebonyi State University, Abakaliki, Ebonyi State Nigeria; 3 Department of Paediatrics, College of Medicine UNEC, Enugu, Nigeria; 4 Department of Paediatrics, College of Medicine, Enugu State University of Technology, Enugu, Nigeria; 5 Department of Community Medicine, College of Medicine UNEC, Enugu, Nigeria

**Keywords:** Willingness, Malaria vaccine, Children, Mothers, Hospitals

## Abstract

**Background:**

There are giant steps taken in the introduction of the novel malaria vaccine poised towards reducing mortality and morbidity associated with malaria.

**Objectives:**

This study aimed to determine the knowledge of malaria vaccine and factors militating against willingness to accept the vaccine among mothers presenting in nine hospitals in Enugu metropolis.

**Methods:**

This was a cross-sectional study carried out among 491 mothers who presented with their children in nine hospitals in Enugu metropolis, South-East Nigeria. A pre-tested and interviewer-administered questionnaire was used in this study.

**Results:**

A majority of the respondents, 72.1% were aware of malaria vaccine. A majority of the respondents, 83.1% were willing to receive malaria vaccine. Similarly, a majority of the mothers, 92.9%, were willing to vaccinate baby with the malaria vaccine, while 81.1% were willing to vaccinate self and baby with the malaria vaccine. The subjects who belong to the low socio-economic class were five times less likely to vaccinate self and baby with malaria vaccine when compared with those who were in the high socio-economic class (AOR = 0.2, 95% CI 0.1–0.5). Mothers who had good knowledge of malaria vaccination were 3.3 times more likely to vaccinate self and baby with malaria vaccine when compared with those who had poor knowledge of malaria vaccination (AOR = 3.3, 95% CI 1–6–6.8).

**Conclusion:**

Although the study documented a high vaccine acceptance among the mothers, there exists a poor knowledge of the malaria vaccine among them.

## Background

Malaria is an infectious disease seen mainly in the tropics. It is transmitted by infected female *Anopheles* mosquitoes [1]. In 2022, there were about 249 million malaria cases globally, and 608,000 malaria deaths in 85 countries, this exceeded the pre-pandemic level of 233 million in 2019 by 16 million cases [1]. Malaria among the under-five in Nigeria is a leading cause of global child mortality, with 95,000 annual child deaths [2]. A household survey conducted in Enugu metropolis noted that more than 50% of the households had an episode of malaria attack within 1 month with an average household expenditure per case noted to be as high as 12.57US$ and 23.20US$ [3]. In Enugu, an estimated malaria cases of 1,177,000 was noted, where 12.9% of children are exposed to insecticide-treated nets (ITN) [4].

Several preventive strategies have been used to control malaria. This includes indoor residual spraying (IRS), the use of insecticide-treated nets, malaria chemotherapy and prophylaxis for pregnant mothers. The complication of malaria remains the major cause of childhood morbidity and mortality in sub-Saharan Africa [2].

Due to the positive outcome of the pilot roll-out of the vaccine in Ghana, Kenya, and Malawi, the WHO has recommended RTS, S/AS01 to be used against malaria infection and in the prevention of *Plasmodium falciparum* malaria in children in endemic areas. The parasite population from which the recombinant protein was derived was the 3D7 clone of strain NF54 [5, 6].

Over the past 5 years, great progress was made in Nigeria in tackling the disease. For instance, more than 25 million children under the age of five have received malaria seasonal chemoprophylaxis (SMC). The country also aimed to extend universal health coverage from five to 25 percent in the year 2025, and to introduce next generation nets and insecticides and finally to support the government on the delivery of a malaria vaccine campaign [7]. Based on the WHO recommendation on malaria vaccine, the vaccine is now approved in Nigeria on April 15th 2023, less than a week after Ghana became the first country to approve the vaccine [8]. The government has used some educational programmes geared towards improving the RTS roll out. This include malaria vaccination campaign through collaborations between health workers, community leaders and traditional and religious authorities [9, 10].

Malaria vaccine is now recommended to be taken on a schedule of four doses in a child who is more than 5 months of age. Presently, over 2.3 million doses of the vaccine, after proven efficacy and safety have been administered in three African countries [5, 6]. A comprehensive study on the pooled coverage of full immunization among children in some parts of Nigeria showed that only 0.6% infants had a complete and timely vaccination [11]. Recent evidence had suggested that vaccine “hesitancy”—a delay in the willingness to accept or refuse vaccine despite its availability, contributed to the low acceptance of the vaccine among Nigerian children [12].

## Problem statement

The development of vaccines against malaria is very necessary to fight drug-resistant forms, which is prevalent in most parts of the tropics of which Enugu state is not an exception. The use of malaria vaccines are the most viable option that could address the burden of malaria infection [13]. The global socioeconomic burden enacted by this disease in Enugu, Nigeria due to increasing drug resistance and poor vector control measures have necessitated the search for a potent and efficient vaccine [13]. A good knowledge of mothers’ attitude to receive malaria vaccine will help in the development, delivery, implementation and possibly payment of the vaccine.

This study aimed to determine the knowledge and willingness to accept malaria vaccine among mothers who present in the children out patients and immunization clinics of nine different hospitals in Enugu metropolis. Besides, it also elicits factors associated with willingness to vaccinate mother and baby with malaria vaccine.

## Methods

### Study design

This was a health facility based cross-sectional study conducted from October 2023 to December 2023.

### Study area

This study was carried out in Enugu metropolis, the capital city of Enugu State, south-east Nigeria. Enugu urban is made up of 3 local government areas namely: Enugu North, Enugu East, and Enugu South. The vaccination coverage in the area of study is high (84.9%) and is comparable with the global average rate of 83% [14].

### Study population

A total of 491 mothers who presented with their children were recruited from the children out patients and immunization clinics of nine different hospitals in Enugu metropolis namely the Enugu State University Teaching Hospital (Parklane) Enugu, Abakpa Nike Primary Health Centre, Anunciation Specialist Hospital, St. Marys Hospital and Maternity, Dental Hospital, Niger Foundation Hospital, Julius Memorial Hospital, Daberechi Hospital, and Hope Hospital. Mothers who gave consent and who came with their children to the facility of study were included in the study while the mothers who were not willing to participate in the study were excluded.

### Sample size estimation

The use of 95% confidence level with 5% precision rate for a population > 100,000 as documented and validated by Israel et al*.* [15] was used to calculate the minimum sample size. This gave a minimum sample size of 490 with ten percent attrition.

### Sampling technique

All the health facilities in Enugu metropolis Enugu state, southeast Nigeria were listed together with the average monthly records of childhood immunization and other children services based on the attendance. Nine health facilities that have the highest attendance for childhood immunization and other services including children out-patient and children emergency services were selected for the study. The respondents were mothers who presented with their babies for immunization or treatment services in any of the children’s clinics. A systematic random sampling technique was used to select the respondents for inclusion in the study. Proportionate allocation of respondents was done based on the average number of mothers who presented in each of the health facilities for the services indicated. Sampling interval for each of the health facilities was determined separately by dividing the sampling frame (i.e., average number of mothers that present in each of the facilities for the services indicated) by the sample size (i.e., number allocated to each health facility based on the proportionate allocation). The index client was selected for each of the health facilities by a simple random sampling technique of balloting.

### Data collection

A pre-tested and interviewer-administered questionnaire was used in this study. This tool has been used by several studies [16–18]. The questionnaire included 3 major models such as the health belief model which assessed domains, such as perceived benefits, perceived susceptibility and perceived barriers while behavioural domain assessed knowledge, attitude and practice. The socio-demographic model used in this study included the gender, age, marital and employment status. The research assistants ensured during the administration of the questionnaire that the respondents responded to all the questions.

### Data analysis

Data entry and analysis were done using International Business Machine, Statistical Product for Service Solutions (IBM-SPSS) statistical software version 25. (IBM Corporation, Armonk, NY, USA). Categorical variables were described using frequencies and proportions while continuous variables were presented using mean and standard deviation. Chi square test and statistical significance and multivariate analysis using binary logistic regression were used in the analysis and the level of statistical significance was determined by a p value of < 0.05.

Outcome variable was willingness to vaccinate Self (the mother) and the baby. In determining the factors associated with willingness to vaccinate Self and baby, the outcome variable was cross tabulated with the socio-demographic characteristics of the mother, the gender of the child and other variable that followed a logical sequence. The variables that had a p value of ≤ 0.2 on bivariate analysis were entered into the logistic regression model to determine the predictor of willingness to vaccinate Self and baby. The results of the logistic regression analysis were presented using adjusted odds ratio (AOR) and 95% Confidence Interval and the level of statistical significance was determined by a p value of < 0.05.

### Socio-economics status of respondents

The use of Principal Component Analysis was applied in the computation of socio-economic status of the respondents [19]. Eight variables were used to determine the knowledge of malaria vaccination among the respondents. For each of the eight variables, a correct response was assigned a score of one and an incorrect answer was scored zero. Respondents who correctly answered ≥ 50% of the eight variables were regarded as having Good Knowledge of malaria vaccination while those who scored < 50% were designated as having Poor knowledge of malaria vaccination.

## Results

Table 1 shows the socio-demographic characteristics of the respondents. The mean age of the respondents was 32.0 ± 8.0 years and the highest proportion of the respondents, 34.0% were less than 30 years.

**Table 1:** Socio-demographic characteristics of the respondents

Variable	Frequency (n = 491)	Percent (%)
Age of Respondents in years		
Mean ± SD	32.0 ± 8.0	
Age of Respondents in groups		
< 30 years	167	34.0
30–34 years	144	29.3
35–39 years	91	18.6
≥ 40 years	89	18.1
Marital status		
Single	51	10.4
Married	440	89.6
Gender of child		
Male	240	48.9
Female	251	51.1
Educational attainment of Mother		
No formal education	12	2.4
Primary education	23	4.7
Secondary education	113	23.0
Tertiary education	343	79.9
Employment status of Mother		
Unemployed	52	10.6
Self-employed	169	34.4
Paid employment	270	55.0
Family socio-economic status		
Low socio-economic class	261	53.1
High socio-economic class	230	46.9

Table 2 shows awareness and willingness to receive malaria vaccine among the respondents. A majority of the respondents, 72.1% were aware of malaria vaccine. A majority of the respondents, 83.1% were willing to receive malaria vaccine. Similarly, a majority of the respondents, 92.9%, were willing to vaccinate baby with the malaria vaccine, while 81.1% were willing to vaccinate self and baby with the malaria vaccine.

**Table 2:** Awareness and willingness to receive malaria vaccine among the respondents

Variable	Frequency (n = 491)	Percent (%)
Awareness of malaria vaccine		
Yes	354	72.1
No	137	27.9
Source of information**	(n = 354)	
Health workers	247	69.8
Radio	143	40.4
Television	131	37.0
Newspaper	110	31.1
Friends	107	30.2
Internet/social media	102	28.8
Parents/family	50	14.1
Community meetings	38	10.7
Posters and banners	37	10.5
Religious gathering	29	8.2
Perceived susceptibility to malaria	(n = 491)	
Yes	398	81.1
No	93	18.9
Aware of someone that died from malaria		
Yes	145	29.5
No	346	70.5
Willingness to receive malaria vaccine		
Yes	408	83.1
No	83	16.9
Willing to pay for malaria vaccine	(n = 408)	
Yes	201	49.3
No	207	50.7
Willingness to vaccinate baby with malaria vaccine		
Yes	456	92.9
No	35	7.1
Willingness to vaccinate Self and baby with malaria vaccine		
Yes	398	81.1
No	93	18.9

**Multiple responses encouraged

Table 3 shows knowledge of malaria vaccination among the respondents. Less than a quarter of the respondents, 20.8% were aware that mothers are a priority group for malaria vaccine.53.8% of respondents felt that they had a good knowledge of malaria vaccination.

**Table 3:** Knowledge of malaria vaccination among the respondents

Variable	Frequency (n = 491)	Percent (%)
Belong to high priority group for malaria vaccine in Nigeria		
Yes (correct)	102	20.8
No	389	79.2
Vaccination is a good measure to prevent diseases		
Yes (correct)	300	61.1
No	191	38.9
Malaria vaccine will be of immense help in the fight against the disease		
Yes (correct)	249	50.7
No	242	49.3
One is expected to take antimalarial even after receiving malaria vaccine		
Yes (correct)	165	33.6
No	326	66.4
All vaccination are beneficial to man except malaria vaccine		
Yes	223	45.2
No (correct)	268	54.6
Side effect of malaria vaccine observed after administration		
Yes (correct)	145	29.5
No	346	70.5
No vaccine developed against malaria		
Yes	255	51.9
No (correct)	236	48.1
Nigeria is not interested in malaria vaccine		
Yes	254	51.7
No (correct)	237	48.3
Knowledge of malaria vaccination		
Good	264	53.8
Poor	227	46.2

Table 4 shows factors associated with willingness to vaccinate Self and baby with malaria vaccine. The respondents who were less than 30 years were about six times more likely to vaccinate self and baby with malaria vaccine when compared with those who were 35 years and above (AOR = 5.7, 95% CI 1.7–19.0). The respondents who belong to the low socio-economic class were five times less likely to vaccinate self and baby with malaria vaccine when compared with those who were in the high socio-economic class. (AOR = 0.2, 95% CI 0.1–0.5). The respondents who perceived themselves as being susceptible to malaria were about 27 times more likely to vaccinate self and baby with malaria vaccine when compared with those who did not consider themselves susceptible (AOR = 26.9, 95% CI 13.2–54.7). The respondents who had good knowledge of malaria vaccination were 3.3 times more likely to vaccinate self and baby with malaria vaccine when compared with those who had poor knowledge of malaria vaccination (AOR = 3.3, 95% CI 1–6–6.8).

**Table 4:** Factors associated with willingness to vaccinate Self and Baby with Malaria vaccine

Variable	Willingness to vaccinate Self and Baby (n = 491)		p value**	AOR (95%CI)***
	Yes N (%)	No N (%)		
Age of Respondents in groups				
< 30 years	163 (97.6)	4 (2.4)	< 0.001	5.7 (1.7–19.0)
30–34 years	104 (72.2)	40 (27.8)		0.7 ( 0.3–1.5)
≥ 35 years	131 (72.8)	49 (27.2)		1
Marital status				
Married	363 (82.5)	77 (17.5)	0.017	1.5 (0.6–4.3)
Single	35 (68.6)	16 (31.4)		1
Gender of child				
Male	198 (82.5)	42 (17.5)	0.426	NA
Female	200 (79.7)	51 (20.3)		
Educational attainment of Mother				
Tertiary education	281 (81.9)	62 (18.1)	0.456	NA
Secondary education and below	117 (79.1)	31 (20.9)		
Employment status of Mother				
Unemployed	39 (75.0)	13 (25.0)	0.451	NA
Self-employed	140 (82.8)	29 (17.2)		
Paid employment	219 (81.1)	51 (18.9)		
Socio-economic status				
Low socio-economic class	178 (68.2)	83 (31.8)	< 0.001	0.2 (0.1- 0.5)
High socio-economic class	220 (95.7)	10 (4.3)		1
Perceived susceptibility to malaria				
Yes	375 (94.2)	23 (5.8)	< 0.001	26.9 (13.2–54.7)
No	23 (24.7)	70 (75.3)		
Aware of someone that died from malaria				
Yes	117 (80.7)	28 (19.3)	0.892	NA
No	281 (81.2)	65 (18.8)		
Knowledge of malaria vaccination				
Good	232 (87.9)	32 (12.1)	< 0.001	3.3 (1.6–6.8)
Poor	166 (73.1)	61 (26.9)		

**p value: bivariate analysis NA Not applicable

***Adjusted odds ratio, (95% Confidence Interval on multivariate analysis)

## Discussion

This work was aimed to elicit the willingness of mothers to accept malaria vaccine for their children and associated factors. The study showed that 83.1% of mothers were willing to receive malaria vaccine for themselves and 92.9% were willing to vaccinate their children. This reportage is at variance with some studies. For instance, Musa-Booth et al*.* [20] in Northern Nigeria, noted the acceptance rate of mothers willing to vaccinate their children with malaria vaccine as 32.3%. The difference between the above study and the current study is the later was done in Southern part of the country. Besides, the study was done before the WHO recommendation of RTS,S and may not follow the guidelines and recommendation therein [20]. Other studies, such as Etokidem et al*.* [21], Abdulkadir et al*.* [22] and Romore et al*.* [23], noted acceptance rate of 87%, 94.5% and 88% respectively. Similarly, in a meta-analysis and systemic review by Sulaiman et al*.* [24], it was reported that the aggregate malaria vaccine acceptance rate was 95.3% [24]. In the general population, the acceptance rate was noted to be 94.4% among mothers [25]. Several countries have documented varying degrees of acceptance rate. For instance, acceptance rate of 97.6%, 94.6% and 92.5% have been reported in Nigeria, Ghana and Tanzania respectively [24]. The differences in acceptance rate may be explained by the various sample size used by the authors. Besides, study population and study site may also play a role in explaining these differences in acceptance rate.

Willingness to accept malaria vaccine and uptake are topical issues in Africa and Nigeria. Grant et al*.* [26] have also noted several challenges with malaria vaccine hesitancy especially during early trials where only 50% of the region in the country implemented the trials due to hesitancy [26]. Furthermore, the high acceptance rate of willingness to receive vaccine in this study is due to the high knowledge (87.9%) of awareness of malaria vaccine availability.

Several factors may explain reasons for vaccine unwillingness/hesitancy as seen in this study. These include mothers having doubts that malaria vaccine will be of immense help in the fight against the disease, mothers believing that one is still expected to take antimalarial even after receiving malaria vaccine, they also believed that there is no vaccine that is even developed against malaria, while they also held to the fact that side effect of malaria vaccine is possible after administration of malaria vaccine.

It is reported in the United States, during influenza pandemics that about one out of five mothers were reluctant to receive vaccine during the influenza era [27, 28]. Some studies have also noted concerns on safety of vaccine, belief on vaccine induced autism, vaccine ingredients, efficacy profile, vaccine’s requirement for multiple injections, and poor level of awareness as factors militating against vaccine uptake among mothers [29–33].

Several measures were proposed to put the issue of hesitancy at bay. For instance, Leask et al*.* [34] have documented the issues of re-evaluating the vaccination decisions with vaccine hesitant mothers over a long time using different discussion groups and tailoring the communication methods based on the mother’s category of vaccine hesitancy [34]. Other groups of mothers who either do not want to accept vaccine immediately or out rightly refused vaccination should be guided cautiously without any use of abusive language [35, 36]. It is also important to note that the complications posed by malaria infection is very common among the under-fives. These children should therefore be vaccinated to curb this menace and reduce morbidity and mortality associated with the illness. Vaccine hesitancy is a serious issue and had raised concerns over child health care and public health workers.

Furthermore, 81.1% of mothers were willing to accept malaria vaccination for their children and themselves as revealed in this study. Children and mothers of child bearing age have the greatest burden of malaria infection with very high morbidity and mortality [37].

A very small proportion of the mothers, 20.8% noted that mothers were in high priority groups for malaria vaccination in Nigeria as they (61.1%) believe that malaria vaccine will be of relevance in the fight against the disease. This finding could be explained by the poor knowledge (53.8%) of malaria vaccine seen in the study. Several factors such as fear about the illness and adults not really showing fatal symptoms of malaria were adduced as possible reasons for this assertion [38–41]. However, worries arising from adverse effects from malaria vaccination are reported as the prevailing circumstances militating for the mothers to vaccinate their children [42–44].

This study revealed that mothers who are married are more likely to accept vaccine than those who are separated or single. Anokye et al*.* [45] noted that mothers who were divorced had higher tendency for vaccine hesitancy. In their study, single women were thrice less likely to accept vaccination compared with the married ones. This study was also in agreement with Barrow et al*.* [46] who noted that married individuals were more likely to get vaccinated than unmarried counterparts [46]. This was further supported by a study that noted that mothers who were divorced or single were thrice less likely to complete immunization when compared with those who were married [47]. These findings could be explained by the fact that married mothers are more in proportion than single women in this study.

This study has shown the fact that mothers who belong to the low socio-economic class were five times less likely to vaccinate self and baby with malaria vaccine when compared with those who were in the high socio-economic class. This can be explained from the fact that poor socioeconomic class is linked with to a higher hesitancy which is also intertwined with less-educated, and low economic security [48]. Low socio-economic class also counters willingness to revive vaccine by altering the interactions between regional economic resilience and spatial connectivity [49]. This could also be explained by the lesser knowledge they have about the vaccine.

Marzo et al*.* [50] and Soma-Pillay et al*.* [51] also documented higher tendency of willingness to vaccinate children among mothers with a higher socio-economic class and education level. The similarity noted in the study above and the current study could be due to similarity in socio-demographic variables. Several studies have shown higher hesitancy among those with lower socio-economic class [48, 52–58].

It is reported in this study that mothers with higher level of education are more willing to accept Malaria vaccine. Studies such as that of Anokye et al*.* [45] have also shown that maternal education plays a vital role in vaccine hesitancy and willingness to accept vaccine. Furthermore an Eritrean study also documented that children of mothers with middle or higher education were 3.2 times more likely to be fully vaccinated than children of mothers with no education [59]. This was also buttressed in a study where 92% of children of mothers with middle or higher education were fully immunized compared with 78% of children whose mothers had no education [60]. Maternal level of education plays a very important role as modifiers of vaccination experiences and behaviours, this helps to put at bay every myth and disbelief on malaria vaccinology [61, 62]. An Indonesian study has shown that acceptance of vaccine was higher in individuals having postgraduate education compared with those with lower education level. This high education levels among mothers with high vaccine acceptance has led to a very high improvement in acceptance and compliance [62]. The findings above were at variance with that seen in the Ethiopia where no association between caretakers’ educational level and vaccination was documented [63]. The current study was undertaken in 9 hospitals drawn from the urban areas of the state and this could explain reasons for these differences.

Mothers who believed they could be infected with malaria vaccine and who had a good knowledge of malaria vaccine were twenty-seven times more likely to receive the vaccine when compared with those who did not agree with such respectively. Besides, mothers who had a good knowledge of malaria vaccine were about three times more likely to receive the malaria vaccine when compared with those who were ignorant of of malaria vaccine. An important factor noted in this study that is strongly associated with willingness to vaccinate both mother and baby with malaria vaccine is knowledge of availability of malaria vaccine as 72.1% of mothers who were aware of the the availability of malaria vaccine are willing to accept the vaccine both for themselves and their children. This may be explained by their inclination to various sources of information on malaria vaccine where information on the malaria vaccine were received principally from health workers, 69.8%; radio, 40.4% and television, 37.0%. The acceptance rate is quite high when compared with that of Musa-Booth et al*.* [20] who noted a acceptance rate of 30% of mothers’ awareness of vaccine in their reportage. They also noted that young maternal age, self-employment and formal employment were linked with poor awareness of malaria vaccine. The high level of awareness from this study could partly be explained by the mothers’ high level of education and socio-economic class. Most importantly is that there is an increased adequate information dissemination on malaria vaccine recently, and this may contribute significantly to the awareness.

The current study also showed that mothers who were less than 30 years were about six times more likely to vaccinate self and baby with malaria vaccine when compared with those who were 35 years and above. The preponderance of young maternal age is corroborated in the reportage of Musa-Booth et al*.* [20] where they noted that mothers who are less than 30 years of age are more willing to accept malaria vaccine. Forty-three percent (49.3%) of the mothers are willing to pay (WTP) for the malaria vaccine. This low figure may be related to the low interest in the malaria vaccine as 51.7% of mothers reported lack of interest in the vaccine as seen in this study. This finding was lower than that of Wagnew et al*.* [63] who reported WTP for childhood malaria vaccine among mothers of under-five children was 60.6%. Higher values have also been reported in Kenya (88%) [25]. The high tendency for WTP for malaria vaccine for the latter studies may be due to the high burden of childhood malaria mortality seen in these countries.

## Limitation

A very large cohort where a very large sample were recruited in the whole country and mothers followed up over a long time may help strengthen this work further.

## Strength of the study

This is the first time this work was executed in t5is setting. Previous works were done in rural areas. Besides, a large sample size and a strong sample frame from where 9 hospitals (mainly primary health centres) were recruited for the study makes the study worthwhile.

## Conclusion

Although the study documented a high vaccine acceptance among the mothers. However, there exists a poor knowledge of the malaria vaccine among them.

## Data Availability

Data are however available from the authors upon reasonable request and with permission of the corresponding author.
